# Effect of High-Dose Topical Minoxidil on Erythrocyte Quality in SKH1 Hairless Mice

**DOI:** 10.3390/ani10040731

**Published:** 2020-04-23

**Authors:** Eduardo Naranjo-Vázquez, María Guadalupe Sánchez-Parada, Belinda Claudia Gómez-Meda, Ana Lourdes Zamora-Perez, Martha Patricia Gallegos-Arreola, Ana Elizabeth González-Santiago, Guillermo Moisés Zúñiga-González

**Affiliations:** 1 Departamento de Ciencias Biomédicas, División de Ciencias de la Salud, Centro Universitario de Tonalá, Universidad de Guadalajara, Tonalá, Jalisco 48525, Mexico; 2 Laboratorio de Mutagénesis, Centro de Investigación Biomédica de Occidente, Instituto Mexicano del Seguro Social, Guadalajara, Jalisco 44340, Mexico; 3 Instituto de Genética Humana “Dr. Enrique Corona Rivera”, Departamento de Biología Molecular y Genómica, Centro Universitario de Ciencias de la Salud, Universidad de Guadalajara, Guadalajara, Jalisco 44340, Mexico; 4 Instituto de Investigación en Odontología, Departamento de Clínicas Odontológicas Integrales, Centro Universitario de Ciencias de la Salud, Universidad de Guadalajara, Guadalajara, Jalisco 44340, Mexico; 5 Laboratorio de Genética Molecular, Centro de Investigación Biomédica de Occidente, Instituto Mexicano del Seguro Social, Guadalajara, Jalisco 44340, Mexico

**Keywords:** hairless mice, micronuclei, genotoxicity, minoxidil, DNA damage, mitogenic agent

## Abstract

**Simple Summary:**

In any animal species, involuntary exposure to unknown agents may increase genetic material damage. This genetic damage can also induce the appearance of diseases such as cancer or other pathologies, including problems that can be passed on to the offspring of the damaged individual. For instance, living organisms may be affected due to the use of medications or exposure to certain chemical, physical, or biological agents which cause cell failure. This impaired function acts as an indicator that helps identify and evaluate damage in order to avoid or minimize it. In this work, excessive doses of a cosmetic drug for topical use in dermatological treatments, known as minoxidil, produced defects in the blood of hairless mice, particularly in red cells, indicating loss of DNA, a situation that may compromise life or the offspring by causing damage to their genetic material. It is important to consider that compounds may be tissue- or species-specific, although we cannot rule out the possibility that similar damage could occur in other animal species. Thus, excessive exposure to this compound should be prevented.

**Abstract:**

SKH1 hairless mice are widely used in carcinogenesis and dermatology research due to their bare skin, as exposure to different agents is facilitated. Minoxidil is a cosmetic drug that is recognized as a mitogenic agent, and mitogens are suggested to have carcinogenic and mutagenic potential by inducing cell division and increasing the possibility of perpetuating DNA damage. Therefore, we hypothesized that the application of high doses of minoxidil to the skin of hairless mice would increase the number of micronucleated erythrocytes (MNEs) in peripheral blood. The objective of this study was to evaluate the topical administration of high doses of minoxidil on peripheral blood erythrocytes of SKH1 mice by means of micronucleus assay. Minoxidil was administered on the entire body surface of mice every 12 or 24 h. Minoxidil dosing every 24 h increased the number of micronucleated polychromatic erythrocytes (MNPCEs), and dosing every 12 h increased the number of MNEs and MNPCEs, as compared to baseline and the negative control group. No decrease in polychromatic erythrocyte frequencies was observed in the minoxidil groups. Therefore, topical application of high minoxidil doses to mice can produce DNA damage, as observed through an increase in the number of MNEs, without producing cytotoxicity, possibly due to its mitogenic effect.

## 1. Introduction

Living organisms are exposed daily to a wide variety of chemical compounds that induce not only acute toxic effects but also alterations in the integrity of their genetic material [1]. These genotoxic agents are commonly used without adequate precautions due to ignorance regarding their deleterious effects and carcinogenic potential. Therefore, there is a need to evaluate the genotoxicity of chemical compounds that are suspected to be responsible for this latent danger [2,3,4,5]. SKH1 hairless mice are widely used in carcinogenesis and dermatology studies due to their bare skin, which favors exposure to different agents [6,7,8,9,10]. In this regard, some mitogenic chemical agents are considered potential carcinogenic and mutagenic agents since they favor the rate of mutation and cell proliferation and thus perpetuate existing damage, favoring the continuity of mutations and the development of cancer, among other mechanisms [11,12,13,14,15]. Although some authors postulate that mitogenic agents are not genotoxic, it is accepted that they are indirectly genotoxic; moreover, this mutagenic activity is suggested to be a secondary event to the cell proliferation induced by these agents [11,14,16].

Minoxidil (C_9_H_15_N_5_O, IUPAC: 6-piperidin-1-ylpyrimidine-2,4-diamine 3-oxide, CAS number: 38304-91-5) is a mitogenic drug used for the treatment of arterial hypertension due to its vasodilator effect, and has also been used as a cosmetic drug since it stimulates hair follicles to promote hair growth [17,18,19]. Although the mechanism of action of minoxidil is still unclear, it is suggested that its vasodilatory effect increases blood flow at the level of the dermal papilla, which favors local irrigation and follicular proliferation of matrix cells [18,20,21,22,23]. The increase in the expression of vascular endothelial growth factor in the anagen phase of follicular growth has also been postulated as a mechanism of action [24], in addition to an increase in the expression of prostaglandin E2 receptor, which contributes to the prolongation of the anagen phase and production of prostaglandin E2 by dermal papilla cells, thus favoring vascular flow [23,25,26,27,28].

The micronucleus test in peripheral blood is a widely accepted and useful assay to determine the effect of agents on chromosomes directly or indirectly [1]. Micronuclei are fragments of chromosomes or whole chromosomes that are situated outside of the nucleus in mitosis because of clastogenic agents (that break chromosomes) or aneuploidogenic agents (that damage the mitotic spindle). An increase in the number of micronucleated erythrocytes (MNEs) indicates that cells originating in the micronucleus present a loss of genetic material [1]. Moreover, increased micronuclei values have been used as a carcinogenesis marker [1,29]. In a previous study, Schop and Goldberg [30] did not observe genotoxicity or cytotoxicity in the bone marrow when topical minoxidil was applied every 24 h for one week using 1% and 5% formulations, although micronuclei values were higher in the exposed group, and an increase in the mitotic index of hair follicle cells was also observed, without being statistically significant.

It is important to highlight that mitogenic agents could be carcinogenic in a dose-dependent manner. In this regard, it would be useful to know whether a higher dose and a longer exposure time could influence these mitogens to exert their possible carcinogenic effect [11,13,14]. Given that the carcinogenic capacity of mitogenic agents is known, in the present study, a high dose of the drug was applied topically to the skin of SKH1 mice every 12 or 24 h for a period of 10 days to evaluate genotoxicity by means of a micronucleus test in the peripheral blood erythrocytes of hairless mice, using a specific stain for nucleic acids. We hypothesized that the application of a high dose of topical minoxidil would increase MNEs in hairless mice.

## 2. Materials and Methods

### 2.1. Animals

This research project was approved by the Ethics and Research Committee of the Health Sciences Division of the Centro Universitario de Tonalá, Universidad de Guadalajara, Guadalajara, Jalisco, Mexico (Registration number: DSC/087/2017). The experiments were carried out at the Centro de Investigación Biomédica de Occidente, Instituto Mexicano del Seguro Social, Guadalajara, Jalisco, Mexico, in accordance with the Provisions for the Use and Care of Experimental Animals (NOM-062-ZOO-1999) and International Health Institutes for the Humane Treatment of Research Animals in order to comply with the Animal Research: Reporting of In Vivo Experiments (ARRIVE) guidelines [31,32].

SKH1 hairless mice were used since this strain is an euthymic and immunocompetent model and is suitable for the evaluation of topical drugs [10,33], allowing the simulation of a zone of alopecia that facilitates the administration of the drug. We used 25 adult male SKH1 mice aged 10 weeks (31.48 ± 3.28 g average weight) supplied by the laboratory animal facility from the Centro de Investigación Biomédica de Occidente, Instituto Mexicano del Seguro Social, Guadalajara, Jalisco, Mexico. The animals (5 mice/group) were kept under standard environmental conditions and housed in polycarbonate boxes in windowless rooms with automatic temperature control (22 ± 2 °C), light control (lights on at 07:00 am–19:00 pm), and maintained relative humidity (50 ± 10%). They were given a standard diet (Purina^®^, St. Louis, MO, USA), including tap water available ad libitum.

### 2.2. Study Groups and Dosage Administration

Five groups were formed: Group 1 (negative control) received 1 mL of double-distilled water through the topical route by means of an atomizer every 24 h for 10 days. Group 2 received a 1-mL vehicle of topical 5% propylene glycol (Reter, Tehuacán, Puebla, Mexico) in 70% hydroalcoholic solution, by spray, every 24 h for 10 days. Group 3 (positive control) received 37.5 mg of 5-fluorouracil (Efudix^®^, Valeant, Brasil) in a 5% gel, which was administered topically every 24 h for a period of 10 days. Group 4 received dose 1, consisting of 5% minoxidil in propylene glycol every 24 h applied topically with an atomizer for 10 days. Group 5 received dose 2, consisting of 5% minoxidil in propylene glycol every 12 h applied topically with an atomizer for 10 days. The applications were performed using a 1-mL spray of the respective solution on the entire body surface of the mouse, making sure that the mouse was totally impregnated. Each mouse was then left separately in a glass container until the minoxidil solution had dried, and then they were housed in their cages.

### 2.3. Slide Preparation and Sample Analysis

The evaluation of the minoxidil was carried out by means of a micronucleus test [34]. Three samples of peripheral blood were taken (at baseline and 144 and 240 h after the first dose). Samples were taken by means of a small excoriation at the tip of the tail to perform smears in precoded microscopic slides in duplicate for each mouse. The samples were air-dried, fixed in absolute ethanol for 10 min, and stained with acridine orange, a specific stain for nucleic acids. The stained samples were analyzed with an Olympus BX51 fluorescence microscope (Olympus, Tokyo, Japan) with a blue fluorescence filter (Olympus DMB-2) and an immersion objective lens (100×).

The parameters analyzed included the number of MNEs per 10,000 total erythrocytes (TEs) in order to assess the damage accumulated during the exposure time, the number of micronucleated polychromatic erythrocytes (MNPCEs) per 1000 polychromatic erythrocytes (PCEs) in order to assess recent damage produced 24 h before sampling, and the proportion of PCEs per 1000 TEs in order to assess cytotoxicity.

### 2.4. Statistical Analysis

Statistical Package for Social Science software v.18.0 (SPSS, IBM Co. Armonk, NY, USA) was used for the analysis of the results, and all data were expressed in 1000 cells (‰) as the mean ± standard deviation. Statistical comparisons were performed with ANOVA and the Bonferroni post hoc test for intergroup comparisons and repeated measures ANOVA and Dunnett’s post hoc test for intragroup comparisons. Statistical significance was indicated by values of *p* < 0.05.

## 3. Results

There were no significant increases in MNEs or MNPCEs in Group 1 (negative control) nor in Group 2 (vehicle) at the different sampling times, and there were no significant differences in the proportions of PCEs.

**Figure 1:**
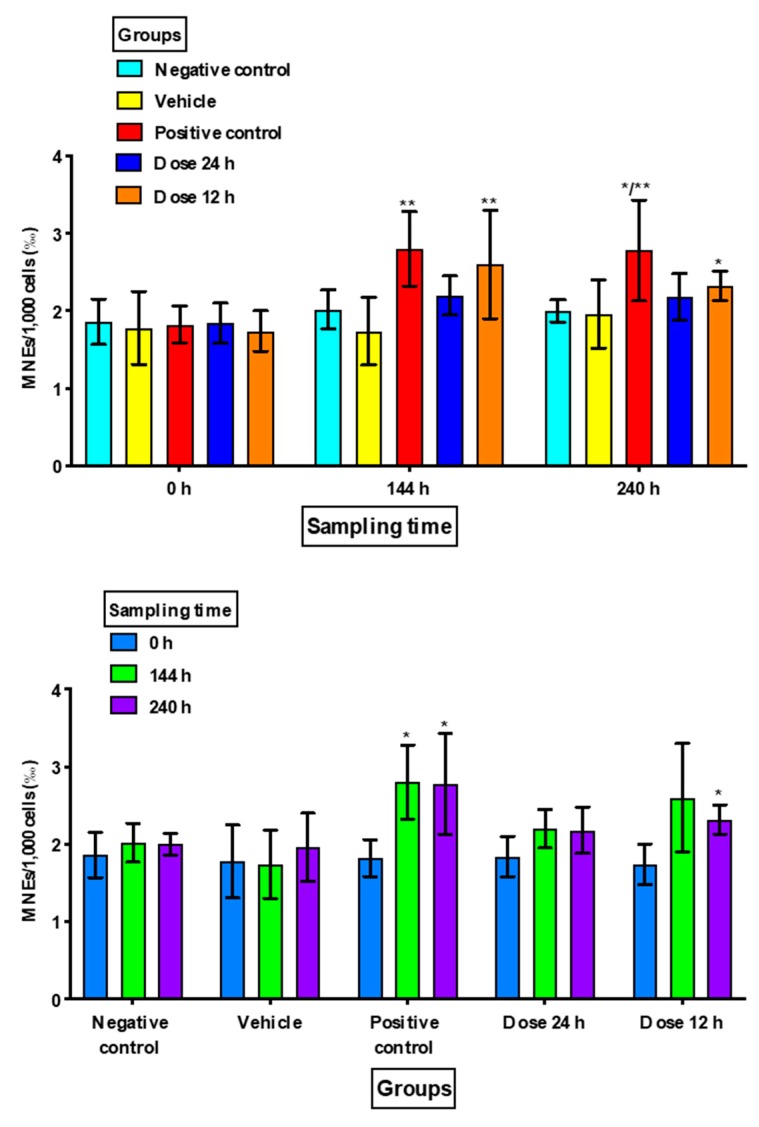
Frequency of micronucleated erythrocytes (MNEs) in the study groups at the different sampling times. Frequency values are expressed as the mean ± standard deviation in 1000 cells (‰). h: hours. Intergroup comparisons were made between the study groups: * *versus* negative control and ** *versus* vehicle. Intragroup comparisons were made between the * basal value (0 h) and sampling times (144 and 240 h).

**Figure 2:**
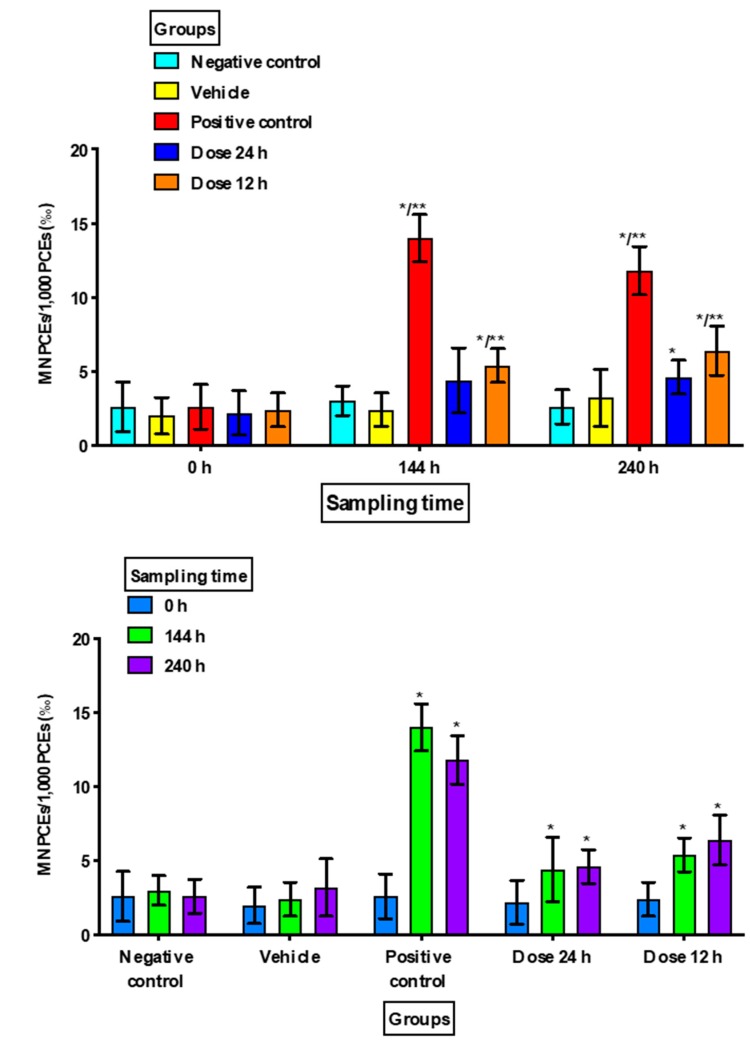
Frequency of micronucleated polychromatic erythrocytes (MNPCEs) in the study groups at the different sampling times. Frequency values are expressed as the mean ± standard deviation in 1000 cells (‰). PCEs: polychromatic erythrocytes; h: hours. Intergroup comparisons were made between the study groups: * *versus* negative control and ** *versus* vehicle. Intragroup comparisons were made between the * basal value (0 h) and sampling times (144 and 240 h).

**Figure 3:**
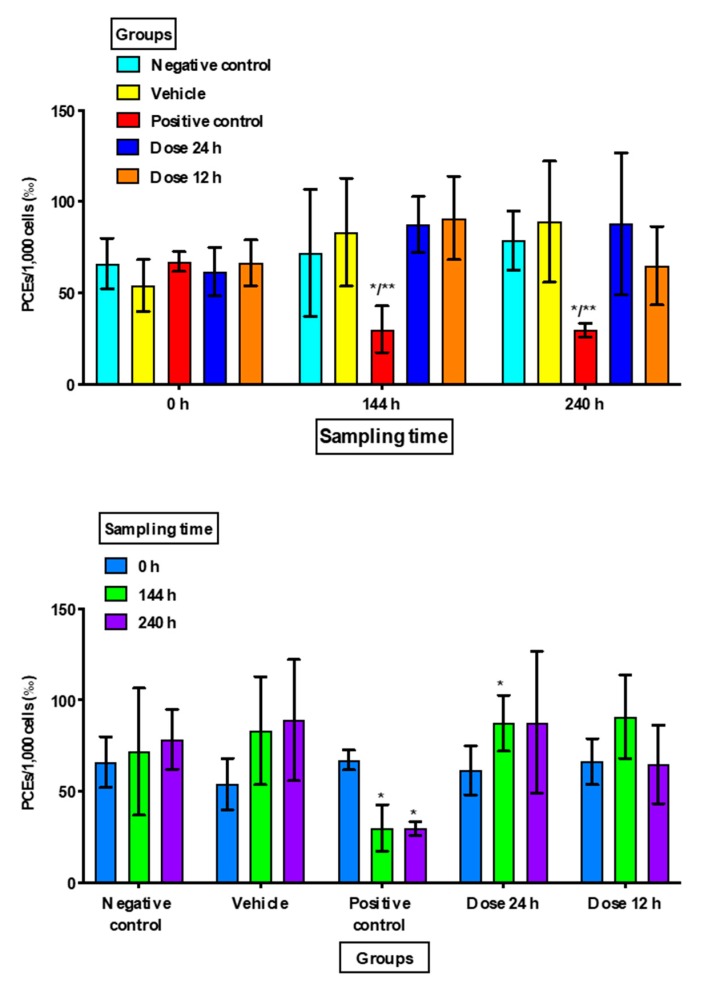
Frequency of polychromatic erythrocytes (PCEs) in the study groups at the different sampling times. Frequency values are expressed as the mean ± standard deviation in 1000 cells (‰). h: hours; NS: not significant. Intergroup comparisons were between the study groups: * *versus* negative control and ** *versus* vehicle. Intragroup comparisons were made between the * basal value (0 h) and sampling times (144 and 240 h).

Group 3 (positive control) showed a significant increase in MNEs at 240 h compared to the negative control (*p =* 0.049) and at 144 h and 240 h when compared to the vehicle group (*p =* 0.016 and *p =* 0.034, respectively). MNPCE frequencies showed a significant increase at 144 h and 240 h (*p <* 0.001) with respect to the negative control and vehicle groups in the intragroup comparison. There was also a statistically significant decrease in PCEs at 144 h and at 240 h as compared to the negative control (*p <* 0.02) and vehicle groups (*p <* 0.03) in the intergroup comparison, and as compared to the baseline value in the intragroup comparison (*p <* 0.005) (Figure 1, Figure 2 and Figure 3).

For Group 4 (minoxidil dose 1/every 24 h), although there was an increase in MNEs compared to the baseline samples, it was not statistically significant, while Group 5 (minoxidil dose 2/every 12 h) showed an increase in MNEs at 240 h (*p =* 0.017) as compared with Group 1 (negative control) and at 144 h (*p =* 0.049) as compared to Group 2 (vehicle). In the intragroup comparison, only Group 5 (dose 2/every 12 h) showed a significant difference at 240 h (*p =* 0.035) as compared with its baseline value (Figure 1).

Regarding the MNPCE frequency (Figure 2), Group 4 (dose 1/every 24 h) showed a difference at 240 h (*p =* 0.033) in the intergroup comparison with Group 1 (negative control), and at 144 h and 240 h in the intragroup analysis as compared with the baseline value (*p =* 0.040 and *p =* 0.004, respectively). Meanwhile, Group 5 (dose 2/every 12 h) exhibited significant differences at 144 and 240 h in the intragroup analysis (*p =* 0.028 and *p =* 0.022, respectively), as well as in the intergroup comparison with Group 1/negative control (*p =* 0.008 and *p =* 0.009, respectively) and Group 2/vehicle (*p =* 0.044 and *p =* 0.036, respectively).

The application of topical minoxidil in both groups (at 24 h and 12 h) did not reveal a statistically significant decrease in the proportion of PCEs as compared with the negative control group, the vehicle group, or the baseline group (Figure 3). However, when minoxidil was applied every 24 h a significant increase was found in PCE frequency (*p =* 0.027) at 144 h in intra-group comparisons. 

## 4. Discussion

The micronucleus test has been widely used to measure the degree of cytogenotoxicity of several chemical compounds; hence, there is a need to evaluate the exposure to harmful agents that are commonly used and affect the lives of organisms [35,36,37,38]. In this study, by means of the micronucleus test in peripheral blood of SKH1 hairless mice, a high dose of topical minoxidil at 5% was analyzed, as this drug has been identified as a mitogenic agent with carcinogenic potential [30,39,40,41] which could be dose-dependent [11,12,13,14,18,42,43,44]. Previous clinical trials established appropriate doses for the effective use of minoxidil for both therapeutic and aesthetic purposes. A high dose of topical minoxidil was used in the present study, as it was applied to the entire body surface of the mouse [18,43,45,46,47,48].

The dose of 5-fluorouracil used for this study as a positive control was based on that of a previous study, where it was identified as being micronucleogenic without being lethal to experimental mice [49]. The genotoxicity presented by 5-fluorouracil in the present study was observed at 240 h, as evidenced by an increase in MNEs and MNPCEs as well as a decrease in PCEs at 144 and 240 h, which are signs of cytotoxicity. The results found with 5-fluorouracil in this study are consistent with previously published results [49]. The negative control and vehicle groups did not show significant differences in the numbers of MNEs, MNPCEs, and PCEs in the samples after the baseline doses, so the manipulated variable was adequately assessed, and it can be ruled out that the observed effect was due to the vehicle.

To ensure a high dose in the application of minoxidil to the mouse, the solution was applied to the entire body of the mouse, ensuring greater compound absorption. One milliliter was sufficient to impregnate the mouse through the body surface; with this, only repetitions of the application during the day could ensure a greater dose. This procedure was performed with the group that received minoxidil every 12 h (twice a day). Results showed that high-dose minoxidil (applied to the full body of the mouse every 12 h), produced a significant increase in micronuclei in the erythrocytes of adults male SKH1 mice, which can be interpreted as damage to the genetic material of the mouse. The objective of including this group was to increase the dose using the same concentration and the same surface area exposed to the compound.

The group in which topical minoxidil was applied once a day did not show a significant increase in MNEs in either of the two samples after the basal sample nor as compared to the negative control or vehicle groups; the group in which topical minoxidil was applied twice a day showed a significant increase in MNEs in the two samples after baseline. These results showed that topical minoxidil applied at high doses and twice a day increased accumulated DNA damage, as observed through the increase in MNEs.

We observed that in both study groups where topical minoxidil was applied once or twice a day there was an increase in MNPCE numbers at 144 h and 240 h as compared to baseline values. These results showed that topical minoxidil every 12 h or every 24 h produced an increase in recent damage to genetic material, as reflected in the increase in MNPCEs. Schop and Goldberg [30] evaluated the genotoxicity of minoxidil in bone marrow by the micronucleus test and analyzed the number of MNPCEs/500 PCEs, finding that values were increased after minoxidil exposure but without being statistically significant [30]. The authors administered topical 5% minoxidil to mice for 8 days only once a day, with an application area of 1 cm^2^ [30]. The application period, the lower dose, and the reduced body area could explain why they did not observe micronuclei or nuclear abnormalities in the histological sections of the follicles. However, we must consider that compounds may be tissue- or species-specific and dose-dependent, and this could explain the findings of other authors [30].

It is worth mentioning that in the work by Schop and Goldberg and the present study there were marked differences, since we found increased damage in the hairless mouse due to the effect of minoxidil. For example, in the previous work [30], minoxidil was obtained from tablets that dissolved in the vehicle. The authors used CD1 mice, which are mice with hair, and these were shaved to apply minoxidil, which was administered in a surface of 1 cm^2^ for 8 days. There were also differences in staining because the authors used Giemsa and light microscopy, and their count used 500 polychromatic bone marrow cells [30].

In the present study, a 5% minoxidil solution was applied, considering that the SKH1 mouse strain has hairless skin and thus has a better chance of absorbing the compound. The administration was over the entire body surface of the mouse. Acridine orange staining was used, which is specific for nucleic acids, and samples were analyzed by fluorescence microscopy to facilitate the identification of micronuclei (bright yellow) and PCEs (reddish-orange). We counted 1000 PCEs of peripheral blood to determine recent damage in the last 24 h, and 10,000 total erythrocytes were assessed for accumulated damage. In the present work, statistically significant differences were observed, possibly because minoxidil was applied for a longer period (10 days), at a higher dose (every 12 h), and was administered over the entire body surface of the mouse, so the effect at the systemic level was more evident. In addition, the cell count was higher, so there was less bias in the readings, allowing us to identify increased numbers of MNEs in the peripheral blood of the mice studied.

Schop and Goldberg [30], observed no increase in either nuclear aberrations in the hair follicle or micronuclei in the bone marrow, but they observed an increase in the incidence of mitosis, which could indicate a mitogenic effect of this compound at a very low dose.

The results obtained in this study showed that minoxidil is not a cytotoxic drug, since no statistically significant decrease in PCE numbers was found; however, minoxidil applied every 24 h resulted a significant increase in PCE frequency, which could be attributed to the properties of mitogenic agents known to promote cell proliferation [11,16,30]. Mitogenic agents represent a significant factor in carcinogenesis, since increasing cell proliferation increases the probability that the genotoxicity present in the cell will perpetuate itself and lead to mutations that can result in development of cancer [14,15,16,50,51]. Authors such as Cohen and Ellwein [11] postulate that mutagenesis is an activity secondary to cell proliferation induced by mitogenic agents [11,14]. Chemical carcinogens can act by inducing mutations and/or altering control of cell growth. Mitogens are group of chemical compounds that alter cell proliferation, and because of this they do not interact with DNA: they are non-genotoxic carcinogens. Also, mutagens can be very effective carcinogens at doses that also induce accelerated cell division, and mutational activity can occur as an event secondary to cell proliferation [16].

We showed that topical minoxidil at high dose acted as a genotoxic agent in SKH1 hairless mice, either due to the dose or by its mitogenic effect, as the results showed a statistically significant increase in the numbers of MNEs and MNPCEs. However, it is still unknown whether minoxidil behaves as a clastogenic or an aneuploidogenic agent. This determination would require the implementation of other genotoxicity tests to corroborate and expand the obtained results [1]. Due to the above and as reported by several authors, we suggest that some mitogenic agents such as minoxidil, may be potential mutagenic and carcinogenic agents, since they act in a dose-dependent manner [11,12,13,14,30,42]. This was seen in the SKH1 hairless mice under the experimental conditions presented here.

It should be mentioned that the use of topical minoxidil at 2% and 5% formulations in cosmetology is FDA-approved [19], with application recommended every 12 to 24 h for a minimum period of 16 weeks, and continuous application for better results [18,43,44,45,46,47,48,52]. Thus, it is important to continue trials using this kind of mitogenic compound to assess the genotoxicity it may produce in other species.

## 5. Conclusions

Under the present conditions, our results showed that a high dose of 5% topical minoxidil increased the number of MNEs and MNPCE in SKH1 mice, resulting in damage to the genetic material, but without cytotoxic effects.
